# Reversible biomass aerogels with flame retardancy and smart elasticity

**DOI:** 10.1093/nsr/nwae449

**Published:** 2024-12-17

**Authors:** Yue Liu, Yiling Sui, Sinan Zheng, Gustav Nyström, Zhihui Zeng

**Affiliations:** Laboratory for Cellulose & Wood Materials, Empa, Switzerland; Key Laboratory for Liquid-Solid Structural Evolution and Processing of Materials, School of Materials Science and Engineering, Shandong University, China; Key Laboratory for Liquid-Solid Structural Evolution and Processing of Materials, School of Materials Science and Engineering, Shandong University, China; Key Laboratory for Liquid-Solid Structural Evolution and Processing of Materials, School of Materials Science and Engineering, Shandong University, China; Laboratory for Cellulose & Wood Materials, Empa, Switzerland; Department of Health Sciences and Technology, ETH Zürich, Switzerland; Key Laboratory for Liquid-Solid Structural Evolution and Processing of Materials, School of Materials Science and Engineering, Shandong University, China

**Figure 1.**
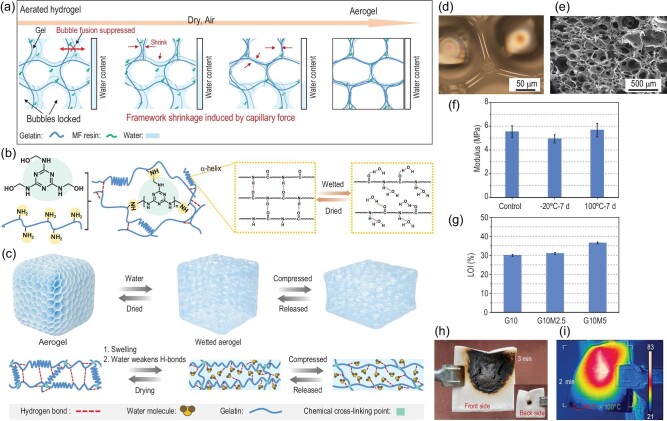
(a) Schematic illustration of structural evolution during ambient-pressure drying processes. (b) Chemistry mechanism behind the fabrication. (c) Schematic diagram of the elasticity transfer of the aerogels. (d) POM images of the aerated hydrogel after being dried at room temperature for 30 min. (e) SEM image of the aerogels. (f) Mechanical stability of the aerogels. (g) LOI values of the aerogels. (h) Digital images of the wetted aerogels after the burning test. (i) Infrared image of the back side of the wetted aerogel burnt for 2 min. Reproduced from ref. [2] with permission.

In materials science, the pursuit of sustainability is increasingly integrated with the drive for innovation [1]. A recent study reported by Yuzhong Wang, Haibo Zhao and collaborators introduces a novel, organic-solvent–free, reversible-gel–assisted, ambient-pressure–dried method for fabricating multifunctional biomass aerogels [2]. This green approach utilizes gelatin, which possesses both thermo-reversible gelling properties and the ability to stabilize air bubbles, as the primary scaffold material. To further enhance the aerogels’ performance, melamine-formaldehyde resin is incorporated as a cross-linking agent and functional component (Fig. 1a–c). The fabrication process involves intense stirring of the polymer solution at elevated temperatures to generate numerous gelatin-stabilized air bubbles. These bubbles are then immobilized within the hydrogel by rapid cooling, followed by drying under ambient pressure and mild conditions (Fig. 1d and e).

This method enables the production of biomass aerogels with remarkable properties, including a high modulus (≤5.5 MPa), low thermal conductivity (30.8 mW m^−1^ K^−1^), and excellent resistance to harsh conditions, such as exposure to various solvents and a wide temperature range from –20°C to 100°C (Fig. 1f). The aerogels also exhibit impressive flame retardancy, with a high limiting oxygen index (LOI) of 36.5% (Fig. 1g), and demonstrate smart, stimulus-responsive behavior in firefighting applications. Notably, the aerogels’ chemical and reversible physical cross-linking structures allow them to undergo unique solvation-controlled transformations. When exposed to water, the aerogel reversibly transforms into an elastic wet gel, absorbing water in the process. This wet gel can withstand continuous external exposure to high-temperature flames (up to 1400°C), maintaining a back temperature of only 80°C, thus providing a safer environment for escape during fires (Fig. 1h and i). These characteristics represent a novel approach to the development of thermal insulation materials for intelligent firefighting applications.

A key strength of this research lies in its ability to combine several desirable properties—flame resistance, elasticity, and mechanical strength—into a single material, addressing the increasing demand for multifunctional and sustainable materials. The authors successfully overcome the challenges associated with ambient-pressure drying, a process that typically leads to structural collapse in traditional aerogels. By addressing these challenges, the study provides a viable pathway for producing aerogels in an energy-efficient manner, while simultaneously improving their performance [3].

In conclusion, Wang and co-workers make a significant contribution to the field of sustainable material design, showcasing the potential of biomass-based aerogels as a versatile solution for a wide range of applications, including construction, automotive, and protective materials [4]. By combining high-strength elasticity with eco-friendly manufacturing methods, these aerogels offer promising solutions to key challenges in material science, with the potential to drive further advancements in the development of sustainable and resilient materials.
